# Current Understanding on Adhesion and Biofilm Development in *Actinobacteria*

**DOI:** 10.1155/2021/6637438

**Published:** 2021-05-22

**Authors:** R. El Othmany, H. Zahir, M. Ellouali, H. Latrache

**Affiliations:** Bioprocess and Bio-Interfaces Team, Faculty of Sciences and Techniques, Sultan Moulay Slimane University, Beni Mellal, Morocco

## Abstract

Biofilm formation and microbial adhesion are two related and complex phenomena. These phenomena are known to play an important role in microbial life and various functions with positive and negative aspects. *Actinobacteria* have wide distribution in aquatic and terrestrial ecosystems. This phylum is very large and diverse and contains two important genera *Streptomyces* and *Mycobacteria*. The genus *Streptomyces* is the most biotechnologically important, while the genus *Mycobacteria* contains the pathogenic species of *Mycobacteriaceae*. According to the literature, the majority of studies carried out on actinomycetes are focused on the detection of new molecules. Despite the well-known diversity and metabolic activities, less attention has been paid to this phylum. Research on adhesion and biofilm formation is not well developed. In the present review, an attempt has been made to review the literature available on the different aspects on biofilm formation and adhesion of *Actinobacteria*. We focus especially on the genus *Streptomyces*. Furthermore, a brief overview about the molecules and structures involved in the adhesion phenomenon in the most relevant genus is summarized. We mention the mechanisms of quorum sensing and quorum quenching because of their direct association with biofilm formation.

## 1. Introduction

**Figure 1:**
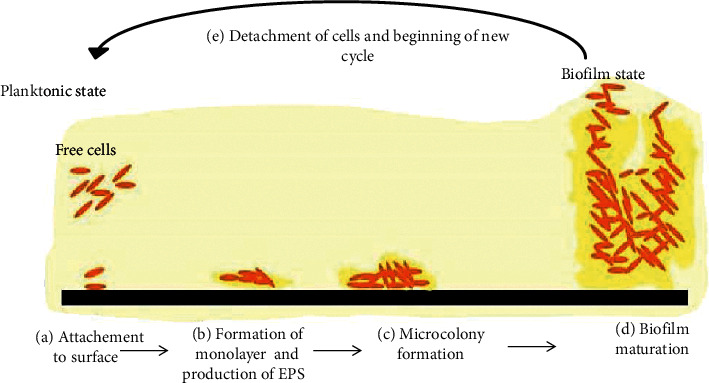
Typical illustration of biofilm formation. The process begins with reversible adhesion of the planktonic cells (brown ovals) with subsequent attachment to the surface (black) (a). The bacteria then form a monolayer and attach irreversibly, inducing an extracellular matrix formation (b). Later, a microcolony is formed where multilayers appear (c). In the following stages, the biofilm matures, forming characteristic structures due to polysaccharides existence (d). At the final step, some cells start to detach and the biofilm (yellow) disperses (e) [2].

A biofilm is a well-organized and collaborative community of microorganisms. These associated cells are differentiated from the suspended ones by reduced growth rate, gene regulation, and the secretion of an extracellular polymer matrix [1]. Biofilm formation undergoes several stages (Figure 1) and involves intracellular signaling and transcription mechanisms distinct from planktonic cells. The biofilm state plays an important role in the community's survival through the protection against various environmental stresses such as pH change, UV radiation, osmotic shock and desiccation, antimicrobial agent penetration, acquisition of new genetic traits, and nutrient availability [3]. This fundamental change in living conditions leads to constant changes in the expression of multiple genes, which causes changes in the morphological, physiological, and biochemical state of cells. Living inside a biofilm provides cells resistance to stressful conditions.

Biofilms occur in several steps: adhesion, maturation, then detachment, and dispersion. The first and most crucial stage is microbial adhesion. Currently, research in the medical field is oriented toward the prevention of microbial adhesion [4–7] and to block quorum sensing [8].

Biofilms are a serious challenge in healthcare-associated infections, especially those involving implantation of medical devices, such as intravascular catheters, urinary catheters, and orthopedic implants [9].

The term biofilm is usually related to negative aspects of microbes association. In many industrial sectors, biofilms represent a real defiance; in addition to cleaning and disinfection problems, they cause energy losses by blocking in condenser tubes, coolers, and heat exchangers. Biofilms affect water quality in drinking water distribution systems by disseminating pathogens [10]. Biofilms can create serious problems in natural, artificial, and biomedical systems [11]. The thing that poses serious problems becomes an obvious advantage in waste treatment and biotechnology. Indeed, cells immobilized within biofilms are adequate in the white biotechnology for production of valuable molecules [12]. Cell immobilization in biofilms can provide specific metabolism in comparison with the suspended culture [13, 14]. Biofilm formation mechanisms are currently the subject of intensive studies and discussions [15].

**Figure 2:**
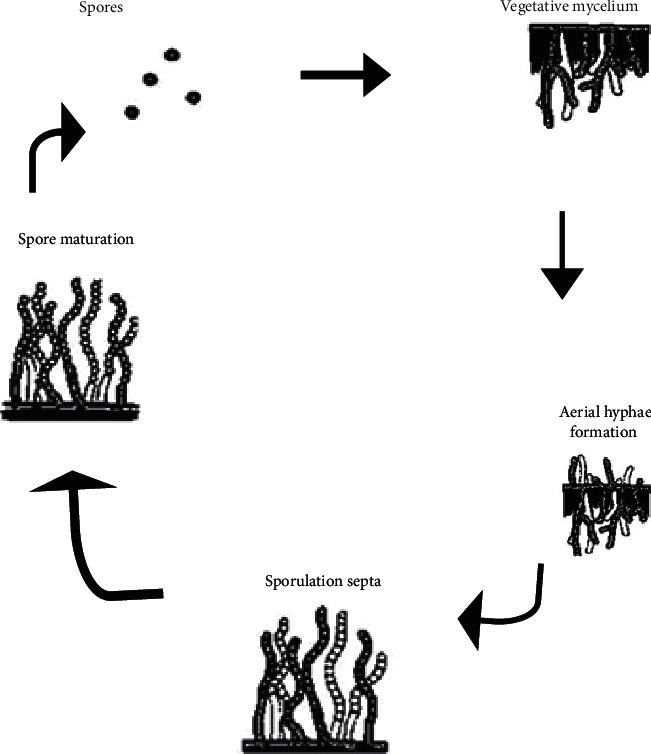
Life cycle of *streptomycetes* [17].

The phylum *Actinobacteria* includes 23 orders, forming one of the largest phyla in the domain bacteria [16]. In December 2015, there were 342 genera with a continuous discovery of other genera. They are typically Gram positive. The life cycle of actinomycetes is unique (Figure 2).

The cycle starts with the germination of “dispersal form” spores and development in branched hyphae named vegetative mycelium, which they differentiate into specialized reproductive structures called aerial mycelium [18]. Considerable effort has been devoted to attack the physiology of bacterial growth and cell division in *Actinobacteria* [19–22]. Despite the abundance of *Actinobacteria*, very few reports were available about biofilm formation in the phylum. This phylum comprises a plethora of phenotypically diverse organisms, with widespread distribution in nature and exhibiting varied oxygen, nutritional, temperature, and pH requirements for growth, making it an important phylum [23]. Their diverse physiological potential makes them a dominant role player in the biotechnology. Their applications are widespread and vary from agroindustry, pharmaceuticals, and bioremediation among numerous others [17, 24, 25]. They play a key role in natural geochemical cycles, especially through their ability to decompose organic matter. *Actinobacteria* are also abundant in the rhizosphere and produce a wide range of biologically active metabolites, thereby influencing plant development [26]. Many *Actinobacteria* are also known pathogens of plants and animals [27]. However, among the most important potential of *Actinobacteria* is the production of a significant number of secondary metabolites like antibiotics and other compounds of biotechnological interest that has been exploited [28].

In addition, members of the genus *Streptomyces* are known to produce more than 70% of commercially available antibiotics [29]. This taxon includes pathologically important strains (e.g., *Mycobacterium tuberculosis* and *Corynebacterium diphtheriae*) and biotechnologically pertinent strains (*Corynebacterium glutamicum* and *Streptomyces griseus*), so exact information on their growth and cell division dynamics is essential to design a treatment or production boosting strategies [18].

Significant information is available on the growth of actinomycetes in shaken-flask cultures and fermenters [30]. Surface growth has received less attention, although surface-associated growth is certainly a different phenotype and a critical stage in their life cycle, with putative morphological and physiological variations that could impact metabolite production [31]. Most of what is known about biofilm formation in *Actinobacteria* comes from the study of antibiotic production in this taxon. While it is indeed the most important phenomenon, the aim of this review is not to present an overview on the biofilm formation mechanisms. The expression of virulence determinants, production of secondary metabolites, and morphogenesis is associated with cell-cell interaction and cell-surface interactions and typically controlled by growth state [32–34]. Further exploration of novel phenotypes under biofilm formation regulations is likely to contribute to the advancement in medical, biotechnological, and ecological fields. Hence, there is a need of studying biofilm formation in *Actinobacteria* especially in *Streptomyces* genus.

In the present work, an attempt has been made to review the literature available on different aspects on biofilm formation and adhesion of *Actinobacteria*. We focus especially on the genus *Streptomyces*. In addition, the literature about the molecules and structures involved in the adhesion phenomenon in the most relevant genus is summarized. We also mention the mechanisms of quorum sensing and quorum quenching; there is a direct relation between these phenomena.

## 2. Biofilm Formation in the Phylum *Actinobacteria*

### 2.1. Importance of Biofilms in Biotechnology

Microorganisms forming monospecific stable biofilms are the most required for their use in industrial processes to produce valuable substances [35]. Many processes are developed to obtain the desired product in bioreactors based on microbial biofilms in the tree levels: laboratory, pilot, and industrial scale. Special designs of “biofilm” bioreactors for culturing microorganisms in biofilm form are described in reference [36]. In fact, the production of organic acids and alcohols and antimicrobial activity are more interesting when the strain is biofilm forming. Various applications in biotechnology using bacterial and fungal biofilms have been published [36, 37]. Recently, environmental biotechnology essentially based on the biodegradation of waste using microorganisms has a great tendency. It is mainly utilized in the processes of industrial wastewater treatment like detoxification and bioremediation and production of electrochemical energy. The application of biofilms for biotechnological purposes has shown great efficiency. Environmental processes based on biofilms are used in the biosorption of heavy metals from wastewater, also for obtaining electrochemical energy from aquatic environments and wastewater [38]. Moreover, in bioremediation and purification of natural environments, the creation of artificial biofilms involving microorganisms gives optimal results and contributes to the resolution of biopurification and bioremediation problems [39]. There is a higher final product yield in bioreactors where a producer is grown as biofilms compared with bioreactors using conventional culture methods; “planktonic cells” occur in the preparation of alcohols—such as ethanol and butanol—and organic acids—such as acetic, lactic, succinic, and fumaric [37, 40]. Qureshi et al. reported that, in bioreactors where *Clostridium acetobutylicum* is grown as a biofilm, butanol production is increased by about 40 to 50 times compared with a conventional bioreactor in a producing batch culture [37]. Moreover, the cellulose production process is reliable and low cost when *Acetobacter xylinum* is grown as a biofilm in a special bioreactor [41]. Acetic acid is produced based on the biofilm culture on industrial scale [36].

With *Streptomycetes* involvement in biofilm formation, there are different approaches. The first approach is co-culture of two organisms—one nonbiofilm former but produces the desired product, and the other is a biofilm producer. This method can optimize the whole process. The second approach is the cultivation of monobiofilms of *Streptomyces* producers. Each of these approaches has technological characteristics and some advantages. The most important tasks of biotechnological biofilms of *Streptomyces* are screening the organism that can actively form a stable biofilm, choosing the conditions of its adherence, selecting an optimal substrate for the fixation and development of biofilms, and studying the parameters of conventional culture (composition of the medium, temperature, agitation rate etc.). For example, the rough surface of the polymethylmethacrylate block as a substrate for adhesion has been found to be more suitable for the formation of streptomycetes biofilms than a smooth surface [40]. Previous studies described different models of bioreactors using biofilms of streptomycetes for laboratory and pilot scales [40]). The production of lactic acid in bioreactors involves the presence of two strains: *Lactobacillus casei* subsp. *Rhamnosus* convert glucose to lactic acid, and *Streptomyces* T7A form stable biofilms. The biofilms formed by *Streptomyces* T7A protect *Lactobacillus casei* subsp. *rhamnosus* from acidity and subsequently contribute to the stability of the process [42, 43].

### 2.2. Current Knowledge on Actinomycetes Adhesion

Attachment is so important in the life of the pathogens of *Streptomyces*. These microorganisms can grow in the liquid-air interface. They can also grow and attach to hydrophobic surfaces such as the leaves of a plant or an animal's skin. Attachment/adhesion could contribute to the effective degradation of substrates by saprophytic streptomycetes. Furthermore, the attachment of microbes to host surfaces is crucial for initiation of infections. Adherence to surfaces is required to establish infections by pathogenic streptomycetes, such as the plant pathogen *Streptomyces scabies* or the human pathogen *Streptomyces somaliensis*. Cell-surface-associated polymers involved in the adhesion of pathogens are unknown because surface polymers have multiple functions in the biology of these organisms [44].

Studying of bacteria to surfaces requires deep knowledge and characterization of both bacterial and substratum surfaces. Research on adhesion of actinomycetes has not received much attention from scientists. The lack of information about this fascinating group comes from the fact that screening for novel molecules with high value is still in progress.

The pathogenic actinomycetes adhesion on medical devices creates a real challenge, so further understanding of the adhesive behavior could contribute to prevention of biofilm formation [45, 46]. Several methods are used to study physicochemical properties and biofilm formation phenomena such as surface hydrophobicity, electron donor character, electron acceptor character, and surface free energy. In physicochemical characterization of the actinomycetes surface, the three pathogenic *Streptomyces* had a hydrophilic cell surface with a weak electron donor character, whereas the surface of *Nocardia* was hydrophobic and strongly electron donor [47].

As a contribution to future research on biofilm formation in soil, actinomycetes were isolated from the saline soil of Beni Amir, Morocco. Bacterial surfaces of actinomycetes were hydrophilic and bipolar at 1 M of salinity. These properties are affected by the salt concentration in the medium [48]. *Thermobifida fusca'*s surface was considered hydrophobic using the MATH method; cell surface hydrophobicity has been implicated in the interactions between cellulosic surfaces. The purpose of investigation is to correlate between attachment and effective degradation of cellulose [49].

## 3. Extracellular Matrix of *Actinobacteria*

Although *Actinobacteria* is one of the largest groups of organisms in the bacterial field, very few reports are available about the biofilm formation in the phylum. An analysis of literature for biofilm formation in *Actinobacteria* revealed that only 9 actinobacterial genera show biofilm ability. Also, limited reports are dissecting the extracellular matrix in some genera [31, 35, 50].

The extracellular polymer matrix of microbial biofilms is a highly complex scaffold. It is characterized by a multitude of structurally and chemically heterogeneous microenvironments. It plays various roles in the structure and function of different biofilm communities. It can be a barrier against adverse chemical and biological influences, such as osmotic stress, acid/base challenges, oxygen, antibiotics and antiseptics, the host immune defense, and grazing protozoa. Moreover, it provides mechanical stability to the biofilm and protects the microorganisms from desiccation and contributes to the sorption and storage of nutrients and trace elements. It is the location of numerous extracellular enzymatic reactions, surface-mediated adhesion, regulatory capacity, and a cohesive network in which the cells are temporarily immobilized. If the biofilm is a microbial city, then the matrix is its infrastructure [3]. Exact and molecular interactions of the extracellular matrix in the biofilm of *Actinobacteria* have not been defined, and the contributions of these components to matrix integrity are poorly understood at a molecular level [51]. The composition of this matrix depends on the species. The extracellular matrix formed by *Streptomyces*—based on building blocks—[44] is morphologically and functionally different from other bacteria. *Streptomyces* form a so-called mycelium that consists of branched filaments that grow by tip extension [52]. This mycelium invades and colonizes the soil environment and secretes numerous enzymes that facilitate break down of plant material, which yields nutrients that can be used as food for the growing mycelium [53].

The transition from vegetative to aerial growth is characterized by a dramatic change in the surface properties of the hyphae. Whereas vegetative hyphae are hydrophilic, aerial hyphae are hydrophobic due to the assembly of an additional outer surface layer or matrix called the rodlet layer [44]. The rodlet layer has an amphipathic nature: the hydrophilic side faces the cell wall, while the hydrophobic side is exposed to the air [54]. The hydrophobic side of the rodlet layer is characterized by thin fibrils. These fibrils are formed by the assembly of so-called chaplin proteins, discovered at the first time in *Streptomyces coelicolor*, providing a hydrophobic character to aerial hyphae [55, 56].

**Figure 3:**
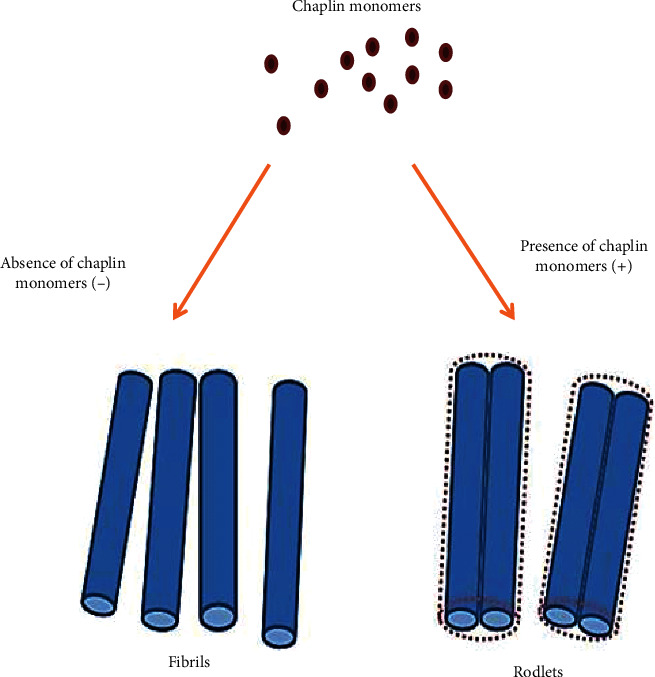
The rodlet layer formed in *Streptomyces coelicolor* strain; it is a result of the assembly of two types of proteins—chaplins and rodlins. In the absence of rodlins, the aggregation of chaplins provides fibrils. This illustration reproduced from Petrus and Claessen's research [44].

Bacterial amyloids have beneficial functions, including conferring stability to biofilms, regulating development, or conferring virulence [57]. For *Streptomyces coelicolor* chaplin proteins are involved in the formation of aerial hyphae, they also mediate attachment to a hydrophobic surface. Chaplin proteins are associated with amyloid-type fibrils to form so-called fimbriae (Figure 3). These fimbriae are anchored to the cell wall via cellulose, providing an important new insight into the role of this polysaccharide in the bacterial domain [58]. The attachment mechanism via cellulose-anchored amyloid fimbriae is widespread in bacteria and may function in the initiation of infection and in the formation of biofilms [58]. Multicellular communities of bacteria are generally connected to each other through a variety of extracellular polymers. These polymers include amyloid fibrils, polysaccharides, lipids, and nucleic acids [59].

Industrial-scale production of important secondary metabolites, such as antibiotics, occurs in large bioreactors. Growth of streptomycetes in such conditions, as opposed to solid-growth or liquid-standing cultures, is characterized by the formation of large clumps, or pellets, consisting of interconnected hyphae [60, 61]. Formation of pellets might be caused by surface-to-surface contact between smaller particles, which as a result can become very large (several millimeters in diameter) [62]. As a consequence, growth occurs predominantly at the outer surface of pellets as oxygen and/or nutrient depletion hampers growth in the central region [60, 63].

Indeed, Manteca et al. indicated that programmed cell death (PCD)-like events occur in the central part of pellets [64, 65]. PCD gives rise to new hyphal growth and also constitutes a serious damage to the integrity of the pellets. In the presence of shear forces in bioreactors, extracellular substances probably contribute to hyphal cohesion. The extracellular DNA (eDNA), which is supposed to be released during hyphal autolysis in the central part of the granules, maintains hyphae together [66, 67]. Similarly, a role for the polysaccharide hyaluronic acid was proposed [66]. More recent evidence indicates that the *CslA*-produced polymer also is involved in pellet architecture [68, 69]. In contrast to the dense pellets formed in the wild-type strain, mycelium of the *CslA* mutant had an open structure [69]. Furthermore, the chaplin proteins were shown to be involved in control of pellet size [68]. Extracellular surface polymers are involved in shaping *Streptomyces* pellets. Interestingly, cell surface polymers might be interesting targets for the industrial improvement of strains, considering the fact that the morphology and size of pellets are important for productivity [61, 68].

## 4. Biofilm Formation by *Actinobacteria*

### 4.1. Streptomyces

The genus *Streptomyces* with 778 species [70] is the largest genus of *Actinobacteria* and is a natural inhabitant of soil and decaying vegetation. *Streptomyces* are widely used to produce useful enzymes and a wide variety of secondary metabolites with potential biological activities. *Streptomyces* produce more than half of clinically useful natural products [71]. They have an inflexible (rigid) cell wall that contains muramic acid with some containing wall teichoic acids. They are excellent producers of antimicrobial secondary metabolites and secrete numerous extracellular enzymes that decompose organic substances [26]. *Streptomyces* is the most studied group of *Actinobacteria* as it is the uncontested and reliable source of many biologically active substances, in particular, antibiotics. Furthermore, the morphological differentiation [72] and exploitation in biotechnology and therapy [73] have received much more attention [18]. However, there are few data available on *Streptomyces* biofilms, but biofilm lifestyle is part of the life cycle of these microorganisms. *Streptomyces somaliensis* and *Streptomyces dangerous* are two species known to be pathogenic to humans and are found in the African region [74]. It has been shown that *Streptomyces* found on devices used in gynecology actively form biofilms in the human body. Three isolates of actinomycetes belonging to the genus *Streptomyces* spp. and *Nocardia* sp. showed a high capacity to form biofilms. Actinomycetes biofilms on the above-mentioned devices are responsible for the development of actinomycosis [50].

Relatively, only few data on the structure of *Streptomyces* biofilms are available yet. In the initial studies on *Streptomyces* biofilms, great emphasis is placed on the ability of these organisms to adhere [58, 75]. *Streptomyces* biofilms are formed on the surface of ancient stone monuments, on cave walls with prehistoric drawings, on old wall paintings and frescoes in historical catacombs, and on the walls of historical and artistic building historic castles in Scotland [76, 77]. A study of 230 biofilms formed on old buildings of different types of materials (cement, lime, concrete, reinforced concrete, brick, and in six European and seven Latin American countries) showed streptomycetes cases are the main biofilm-forming microorganisms on building surfaces.

### 4.2. Surface-Active Proteins Involved in the Attachment of Hyphae to Surfaces and Aerial Growth

Microbial adhesion depends on surface properties of both bacteria and support [7, 78]. Here, we presented the majority of proteins expressed in the cell surface of *Streptomyces*, which mediate attachment. The most of what is known about surface-associated proteins in Streptomyces comes from studies completed on the most pertinent species *Streptomyces coelicolor*. Streptomycetes are capable of adhering to a variety of surfaces, which could be instrumental for invasive growth or for the efficient degradation of substrates, for example, dead plant material. Also, attachment could be important for establishing infections by plant pathogenic streptomycetes, such as *Streptomyces ipomoeae* or *Streptomyces scabies*. Several proteins are constituents of the cell surface of *Streptomyces* involved in their attachment to surfaces.

*Streptomyces* have a complex life cycle, its starts when spore senses a suitable source of nutrients. The germinating spore develops one or two tubes that elongate by apical tip extension. The hyphae form septal cross walls without compartmentation called vegetative mycelia. Aerial hyphae are formed only on solid-growth culture giving colonies their characteristic white and fluffy appearance [52]. The transition from vegetative to aerial growth coincides with a dramatic change in the cell surface properties of the hyphae; whereas vegetative hyphae are hydrophilic, aerial hyphae are characterized by their high surface hydrophobicity [79]. This hydrophobicity is due to the formation of an extracellular proteinaceous surface layer known as the rodlet layer. The rodlet layer is composed of two classes of proteins, chaplins and rodlins [79]. The chaplin proteins in *Streptomyces coelicolor* comprise a class of eight different members, ChpA-H, which can be divided into two groups. The long chaplins, ChpA-C, are about 225 amino acids in length, while the short chaplins, ChpD-H, are considerably smaller (approximately 63 amino acids). ChpD-H consists of a signal sequence for secretion followed by a relatively hydrophobic, so-called chaplin domain. ChpA-C contains two chaplin domains followed by a stretch of hydrophilic amino acids and a cell wall-anchoring domain [55, 56]. Two of the short chaplins, ChpE and ChpH, are secreted in the medium well before aerial growth commences [55].

These chaplins are thought to facilitate aerial growth by lowering the water surface tension, allowing hyphae to breach the medium-air interface. Subsequently, aerial hyphae secrete all chaplins (ChpA-H), which jointly assemble into small fibrils that cover the hyphal surface. Thereby, these aerial structures become hydrophobic. In most streptomycetes, these chaplin fibrils are organized by the rodlin proteins into rodlets [79]. However, this organization only appears to be critical for aerial growth under osmotic stress conditions [80]. Fibrils forming the rodlet layer are often aligned into pairs called rodlets. This process is mediated by the activity of the rodlin proteins RdlA and RdlB [75]. The chaplin protein fibrils provide not only surface hydrophobicity but also rigidity to the aerial hyphae. Notably, three chaplin proteins appear to be invariably present in streptomycetes, which are ChpC, ChpE, and ChpH [81]. ChpE and ChpH are secreted into the environment to reduce surface tension, thereby allowing the hyphae to escape from the aqueous environment into the air. These proteins therefore make it possible to overcome this high-surface-tension barrier [55]. In vitro experiments indicated that chaplins can reduce the surface tension, within minutes, from 72 to 24 mJ m^−2^, making them among the most potent natural surfactants known [55]. Mixtures of cell-wall-extracted chaplins can be used to modify a variety of hydrophilic and hydrophobic surfaces in vitro, thereby changing their nature. Assembly on glass leads to a protein coating that makes the surface hydrophobic. The assembly of chaplin proteins on hydrophobic surfaces make them hydrophilic [82].

In so-called liquid static cultures, strong attachment of *Streptomyces coelicolor* is observed coinciding with the formation of an extracellular matrix that is relatively distinct from the rodlet matrix identified on aerial hyphae [58, 83].

The attachment-associated matrix is characterized by fimbriae structures that appear to be connected to the hyphal surface via protrusions present along the adhering hyphae. Formation of fimbriae is dependent on the presence of the chaplin, inferring that fimbriae are, at least partially, composed of these proteins [58]. Indeed, without chaplins, fimbriae are no longer formed, which concomitantly also prevents hyphae from adhering to the surface.

Similar results were obtained with a mutant strain lacking the *bldN* gene, which encodes an extracytoplasmic function sigma factor required for the expression of the chaplin genes [84, 85]. To mediate attachment to hydrophobic surfaces, fimbriae probably require partial unfolding or unwinding to expose the hydrophobic sides of the individual chaplin fibrils, which are expected to be buried in the interior of the hydrophilic fimbriae [59]. Notably, also adhesion of streptomycetes involves a cellulose-like glycan, which is synthesized by the cellulose synthase-like protein CslA [58]. Attachment of hyphae was significantly reduced in mutant strains lacking the corresponding *cslA* gene. Instead, the glycan was shown to be important for proper anchoring of the fimbriae to the adhering hyphae. SapB is another macromolecule that is secreted during development [86]. This lantibiotic-like peptide [87] lowers the surface tension of the aqueous environment to enable hyphae to grow into the air [88].

### 4.3. *γ*-Butyrolactones as Inducers in Actinomycetes

Quorum sensing or cellular communication plays an important role in the attachment and biofilm formation [89–91]. Intracellular communication takes place through molecules that can be diffused in the surrounding environment. The communication system is based on signal molecules and transcription-activating proteins. In response to a cellular concentration, the signal molecules activate the target gene, which is in conjunction with the LuxR transcription activators. The signal molecules are known as acylhomoserine lactones HSL that are Gram positive. There are other types of signal molecules that are called AIP autoinducer peptides that exist in positive Gram. *Streptomyces* have butanolide-type molecules called gamma-butyrolactones that regulate the production of secondary metabolites such as antibiotics. Nowadays, there are no studies showing the role of autoinductors in biofilm formation in *Streptomyces* [23]. On the other hand, there are some reports about the involvement of signal molecules in biofilm formation in *bifidobacterium*. The autoinducers AI-2 are implicated in the regulation of biofilm formation in *Bifidobacterium* and *Propionibacterium* genera [92, 93]. AI-2 autoinducers are involved in the upregulation of virulence factors [92]. GBLs, MMFs, Factor-A, Factor-I, IM-2, VB, and PI factor are signal molecules regulating quorum sensing in the genus *Streptomyces*. These molecules control the following phenotypes: antibiotic production (Act Red, clavulanic acid, cephamycin, D-cycloserine, Kas, methylenomycin, natamycin, nikkomycin, nucleoside, pristinamycin, streptomycin, tylosin, virginiamycin), morphogenesis, and sporulation.

## 5. Antibiofilm Activity/Compounds from *Actinobacteria*

With constant rise in the number of antibiotic-resistant bacteria, there is a need to find alternative strategies to combat the phenomenon. Since pathogenicity is related to the biofilm formation ability, antibiofilm activity has become the trendiest alternative target to control their spread. Biofilms are intrinsically resistant to conventional antibiotics [94,95]. This indicates the need for new antibacterial drugs active not only against planktonic bacteria but also against drug-resistant biofilms. Various bioactive compounds have shown antibiofilm activity against Gram-positive pathogens [96–98]. The discovery of novel compounds is still very important.

Actinomycetes predominantly account for the production of the majority of bioactive compounds [99]. The antibiofilm activity of actinomycetes can be manifested by several mechanisms: by inhibiting microbial adhesion [100, 101] or by targeting quorum sensing [102, 103]. *Streptomyces akiyoshinensis* (A3) had shown an antibiofilm activity against *S. pyogenes* biofilms. Application of extracts significantly prevents biofilm formation up to 60–80%. Five extracts obtained from *Streptomyces akiyoshinensis* (A3) reduced the cell surface hydrophobicity, which is a crucial factor for biofilm formation in *Staphylococcus pyogenes* [104]. *Streptomyces lunalinharesii* strain 235 produces an active antimicrobial substance against sulfate-reducing bacterial biofilms, a significant problem for the oil industry. Results showed that the supra-minimal inhibitory concentration significantly reduced biofilm formation. The application of this antibiofilm substance as a potential biocide to control the growth of sulfate-reducing bacteria could be of great interest to the oil industry [105]. *Streptomyces chrestomyceticus* strain ADP4 inhibits biofilm formation [101]. The antibiofilm activity exerted by *Streptomyces chrestomyceticus* strain ADP4 against *Candida* spp. had a strong inhibitory effect on *Candida* cells adhesion and subsequent conversion to the hyphal state. These metabolites produced by *Streptomyces chrestomyceticus* strain ADP4 showed strong anti-*Candida* activity. They could be a potential drug to treat biofilm-mediated infections because they prevent cell transformation to biofilms [101] (Table 1).

Balasubramanian et al. evaluated the potential of marine sponge-derived actinomycetes in inhibiting biofilm formation of *Staphylococcus epidermidis*, *Staphylococcus aureus*, and *Pseudomonas aeruginosa*. Results from in vitro biofilm-formation assays and scanning electron and confocal microscopy revealed that an organic extract derived from the marine sponge-associated bacterium *Streptomyces* sp. SBT343 significantly inhibited staphylococcal biofilm formation on polystyrene, glass, and contact lens surfaces without affecting bacterial growth [108].

## 6. Conclusion

*Actinobacteria* is a large phylum [109]. It comprises genera of particular importance in biotechnology and other pathogenic species [110]. However, few reports describe biofilm formation in this phylum [35, 50]. Great efforts are being deployed in areas related to the production of valuable compounds and antibiotics. The biotechnological processes are more efficient using biofilms than planktonic cells. The greatest metabolic and phylogenetic diversity in *Actinobacteria* provides an exceptional opportunity to explore their multifactorial abilities for biotechnological applications [23]. Some studies have focused on the adhesion of *Streptomyces* used in biotechnology. Biofilm steps are still unknown in *Actinobacteria*. The comprehension of biofilm formation in this bacterial group could contribute to several advances, so this issue must not be neglected. To our knowledge, the investigation of biofilm formation in *Actinobacteria* remains poorly explored. The mechanisms can be used in the application of biotechnology research. Future research should be oriented to the extracellular matrix, which constitutes a key factor in the integrity and stability in biofilms. Biofilm formation is an important phenomenon that is underexplored in this phylum.

## Figures and Tables

**Table 1:** Studies conducted on the antibiofilm activities of *Streptomyces* species.

Strains	Molecule	Reference
*Streptomyces akiyoshinensis* (A3)	Antibiofilm activity against *S. pyogenes* biofilms	[104]
*Streptomyces* sp.	Bioactive compound from actinomycetes for antibiofilm activity against Gram-negative and Gram-positive bacteria	[106]
*Streptomyces gandocaensis* strain DHS334	Cahuitamycin C showed highly effective inhibition effects on the biofilm formation of *Acinetobacter baumannii*	[107]
*Streptomyces lunalinharesii* strain 235	Biofilms of sulfate-reducing bacteria	[105]
*Streptomyces chrestomyceticus* strain ADP4	Strong anti-*Candida* activity	[101]
*Streptomyces* sp. SBT343	Marine sponge-derived actinomycetes	[108]
